# Factors Associated with School Nurses’ Triage Competency in South Korea

**DOI:** 10.3390/ijerph18168279

**Published:** 2021-08-05

**Authors:** Jaehee Yoon, Heesook Son

**Affiliations:** 1 Wolchon Elementary School, 132, Mokdongjungang-ro, Yangcheon-gu, Seoul 07980, Korea; rnyjh@snu.ac.kr; 2 Red Cross College of Nursing, Chung-Ang University, 84 Heukseok-ro, Dongjak-gu, Seoul 06974, Korea

**Keywords:** triage competency, school nurse, hospital nurse experience, school nurse experience

## Abstract

This study examined the factors associated with triage competency among school nurses in South Korea. Using a convenience sampling method, 386 school nurses employed in elementary, middle, or high schools completed a cross-sectional survey that included a modified version of the Triage Competency Scale for emergency room nurses. Information regarding experience working in schools and hospitals, education level, school types, age, emergency nursing care certifications, school locations, and serious emergency experience at school was collected. Analyses were performed using SPSS version 25.0, independent *t*-tests, analyses of variance, Spearman’s correlation, and ordinal logistic regression. Triage competency was higher for school nurses who were employed in metropolitan regions (odds ratio [OR] = 1.63, *p* = 0.017) and had serious emergency experience (OR = 1.76, *p* = 0.008). As the participants’ experience at schools or hospitals increased by one year, their triage competency score increased by 2% (OR = 1.02, *p* = 0.037) and 14% (OR = 1.14, *p* < 0.001), respectively. These findings could be used to develop policies and educational programs that promote school nurses’ triage competency. Further, they suggest the importance of establishing an organizational support system to develop guidelines and a feedback system to improve school nurses’ triage competency.

## 1. Introduction

The definition of triage is *to sort or sift*, and it is primarily used in emergency medicine [1,2,3], where it refers to the process of classifying patients according to their symptom severity and medical needs [2]. Before an accurate diagnosis is made, the severity of the medical emergency is classified through rapid assessment of the patient while providing the appropriate emergency medical services. Due to the overcrowding of emergency rooms, efficiently allocating limited emergency resources while providing the safest care to emergency patients remains a critical issue [4,5].

Schools are places where emergencies occur frequently, and a variety of individuals within the school, including students, teachers, and staff, visit the school nurse’s office for their health problems, such as acute conditions, chronic diseases, and accidents [6]. According to the Korean national education statistics [7], the number of visitors to the school nurse’s office has increased annually, with an average annual number of visitors (at elementary, middle, high, and special schools) of 3689 in 2017, 3789 in 2018, and 3967 in 2019. Furthermore, the frequency of visits per student to the school nurse’s office increased from 7.9 in 2017 to 8.5 in 2018 and 9.2 in 2019 [7].

In an emergency, the school nurse evaluates the severity of the situation through rapid assessment and coordinates the necessary procedures at the appropriate level of emergency medical services. In this regard, schools also act as emergency practice sites. As the only medical personnel in the school, the school nurse plays an important role in classifying the severity of a medical emergency with respect to triaging. Due to the limited medical resources that are available to schools, the school nurse’s work is critical to assess emergencies and provide appropriate medical services, including referrals.

A school nurse’s ability to triage is important in terms of the students’ health, efficient use of emergency medical resources nationwide, and students’ academic achievements, because accurate triaging by the school nurse contributes to reducing unnecessary student absenteeism [8]. However, triaging is a very difficult task [9]. Furthermore, studies have demonstrated that Korean school nurses experience difficulties with assessing patients and judging the severity of their conditions; thus, they tend to overclassify patients [10].

School nurses’ triage competencies must be guaranteed and maintained through ongoing training and evaluation [11], highlighting the importance of developing qualification requirements for triage competency when hiring school nurses. Furthermore, it is imperative that continuing education programs that effectively maintain and improve school nurses’ triage competency be developed. However, there is a lack of research around school nurses’ triage competency that could serve as the basis for creating such policies and programs. For the past decade, triage systems could only be found in the School Nurse Emergency Care Course Manual of the Illinois Emergency Medical Services for Children in the United States [12]. Although some research has been conducted on emergency nurses’ triage competency [5,13,14], little is known about the school nurses’ triage competency. Therefore, this study aimed to identify the factors related to triage competency among a sample of school nurses.

## 2. Materials and Methods

### 2.1. Study Design

This descriptive cross-sectional study aimed to determine the factors that influence the school nurses’ triage competency in South Korea.

### 2.2. Setting and Data Collection

The study was conducted in South Korea. Using convenience sampling from members of the Korean Health Teachers Association, 386 school nurses who were employed at elementary, middle, and high schools were recruited to participate in this cross-sectional study. Participants were asked to complete a questionnaire survey. Before the data were collected, the study protocol was approved by the Institutional Review Board (IRB) at the first author’s institutional affiliation (IRB No. 1809/003-009). Details regarding recruitment and data collection were described in a prior paper [15]. Using G-Power version 3.1 (Heinrich-Heine-Universität Düsseldorf, Düsseldorf, Germany), the power was calculated at β = 0.99 with a sample size of 386, α = 0.05, 10 predictors, and medium effect size of 0.15.

### 2.3. Instruments

#### 2.3.1. The Triage Competency of School Nurses

A modified version of the Triage Competency Scale for emergency room nurses that was developed by Moon and Park was used to measure the school nurses’ triage competency [16]. The scale included 5 subdomains: Clinical Judgment (13 items); Expert Assessment (4 items); Management of Medical Resources (4 items); Timely Decisions (4 items); and Communication (5 items). The total score for the 30-item scale ranged from 0 to 120, with each item rated on a 5-point Likert scale. Higher scores indicated higher severity classification competency. In the previous study [15], the nine items that did not fit the school conditions were modified before participants were included in the study. When the scale was developed, Cronbach’s alpha was 0.91, while it was 0.98 in the current study.

#### 2.3.2. Years of Experience and General Characteristics

Two types of work experience were evaluated by asking the respondents about their duration of work experience as a school nurse and their clinical experience prior to becoming a school nurse. The general characteristics included the education level, type of school employed, and location. Questions regarding other factors that were demonstrated as related to triage competency in emergency nursing were asked, including age [17], emergency nursing care certifications [18], and serious emergency experience [19].

### 2.4. Ethical Considerations

This study used the same data that were used to develop an emergency nursing competency scale for school nurses [15]. The study was conducted after it was reviewed and approved (IRB No. 1809/003-009) by the IRB to which the first author is affiliated.

### 2.5. Data Analysis

Data were analyzed using IBM SPSS software version 25.0 (IBM Corp., Armonk, NY, USA) and R-3.3.3 program (The R Project for Statistical Computing, R Core Team, Heinrich-Heine-University, Dusseldorf, Germany). The means, standard deviations, frequencies, and percentages for the participants’ work experience and general characteristics were calculated. To determine the differences in the triage competency according to the participants’ work experience and general characteristics, independent sample *t*-tests and one-way analyses of variance were performed using the least significant difference test for the post-hoc analyses. The correlations between triage competency and nursing experience at schools and in hospitals were analyzed. As a normal distribution could not be assumed with the Kolmogorov–Smirnov and Shapiro–Wilk test, Spearman’s correlation calculations were utilized. Triage competency was divided into three groups based on the total score (i.e., Low Competency for the bottom 25%; High Competency for the top 25%; and Middle Competency for the remaining 50%), and ordinal logistic regression analyses were used to examine the associations between the factors and triage competency levels.

## 3. Results

Table 1 shows the participants’ general characteristics and triage competencies. The mean age was 41.09 years (standard deviation [*SD*] *=* 10.08). On average, the duration of work experience as a school nurse was 10.28 years (*SD =* 9.80), while the duration of hospital experience prior to working as a school nurse was 4.80 years (*SD* = 4.38). A total of 162 (42.0%) participants had experience with a serious school emergency. The triage competency was statistically higher in school nurses who had higher educational levels (χ^2^ = 6.99, *p* < 0.001), worked in metropolitan regions (χ^2^ = 8.44, *p* =0.003), had serious emergency experience (χ^2^ = 3.93, *p* < 0.001), and had certifications related to emergency nursing care (χ^2^ = 2.65, *p* = 0.009). Additionally, the triage competency was higher in older individuals (*F* = 23.2, *p* < 0.001) and in those with more work experience at schools (*p* = 0.018) and hospitals (*p* < 0.001).

Table 2 presents the correlations between the Triage Competency Scale subdomains and work experience. Significant positive correlations were noted when the total Triage Competency Scale scores and the duration of nursing experience at schools (*rho* =0.19, *p* < 0.001) and in hospitals (*rho* = 0.25, *p* < 0.001) were compared. All the Triage Competency Scale subdomains significantly correlated with the duration of experience working as a nurse at schools and in hospitals. In particular, the subdomains of Timely Decisions and Clinical Judgment were highly correlated with the duration of experience as a hospital nurse (*rho* = 0.26, *p* < 0.001 and *rho* = 0.25, *p* < 0.001, respectively). However, the subdomains of Timely Decisions and Clinical Judgment correlated poorly with the duration of experience as a school nurse (*rho* = 0.14, *p* = 0.005 and *rho* = 0.16, *p* = 0.002, respectively).

The factors identified as significant in the univariate analysis were then included in the ordinal logistic regression analysis to examine which factors predicted group membership according to triage competency. As scores that had a variance inflation factor greater than 2.5 may have resulted in multicollinearity [20], we excluded the variable of age (variance influence factor = 4.98) in the regression analysis. Workplace location, serious emergency experience, and experience in both the school and hospital settings were significantly associated with triage competency (Table 3). The triage competency was higher for school nurses who were employed in metropolitan regions (OR = 1.63, *p* = 0.017) and for those who had serious emergency experience (OR = 1.76, *p* = 0.008) compared to the participants who worked in non-metropolitan regions and who did not have serious emergency experience, respectively. As the duration of experience as a school nurse and as a hospital nurse increased by one year, the triage competency increased by 2% and 14%, respectively. Education level and emergency nursing care certifications were not associated with triage competency.

## 4. Discussion

As school nurses are the only medical personnel within the school, their triage competency is a critical component of students’ and school staff’s health. Previous studies have discussed triage competency in emergency nurses; however, little is known about school nurses’ triage competency. Work experience has been demonstrated as an essential component of triage competency [11,13]. In this study, school and hospital nurse experience were factors that influenced triage competency. This finding was consistent with those of previous studies that reviewed emergency room nurses [14,21,22]. These nursing experiences could positively influence triage competency for several reasons. First, this phenomenon could be related to a nurses’ core capabilities when performing in any practice, including triage competency. Nursing competence is defined as the ability acquired as a nurse [15,23]. Triage competency is also considered a type of nursing competency in nursing practice [16]. The Triage Competency Scale subdomains that were evaluated in this study did not deviate from the attributes that were related to nursing competency. Studies demonstrated that nursing competency improved with nursing work experience. The positive factors that develop nursing competency include creating experiences that integrate knowledge and skills in clinical practice, reflecting on the nursing outcomes, and continuing education and training programs while employed [23,24]. These factors accumulate as work experience grows. Additionally, effective communication and teamwork [3,25,26], which are considered attributes of triage competency, are enhanced through a variety of nursing careers and experiences [24]. That is, the core capabilities that are required to develop nursing competency are improved through any nursing work experience, regardless of the differences in the nursing practice. This leads to improvements in overall triage competency.

Second, the relationship between nursing experience and triage competency could be explained by the “clinical acumen” that is required in the triage decision-making process. Experienced nurses use “clinical acumen” more often than novice nurses [2,27]. As clinical acumen is required for the immediate evaluation of a patient’s health condition when a nurse first meets a patient, it is used as part of decision-making strategies through mechanisms such as intuition and deductive reasoning [2]. Further, clinical acumen is developed through knowledge accumulated over an extended period of work or through various practical experiences [27]. Therefore, increased nursing experience could contribute to improved triage competency.

Specifically, we found that the odds ratios and correlations were more significant for the duration of hospital experience than for the duration of experience as a school nurse, when considered in relation to triage competency. Experience as a school nurse provides practical opportunities to care for individuals during emergencies and triage to determine the severity of the emergency. Hospital nurse experience can help develop the core capabilities required to perform as a nurse in any field [15]. Compared to hospital nurse experience, school nurse experience demonstrated greater similarities with emergency room or triage nurses. However, the current findings revealed that hospital nurse experience was of greater importance than school nurse experience that directly involved triaging. This was inconsistent with previous study findings. Martínez-Segura et al. [25] found that nursing experience in the emergency department was the only factor that contributed to triage competency, whereas Chen et al. [21] reported that duration of work as an emergency nurse was associated with triage accuracy. These differences could be related to the lack of organizational factors in schools compared to hospitals. Well-developed guidelines and appropriate feedback, teamwork, and continuing education are organizational factors that influence triage competency [2,26,28]. In particular, nursing experience could negatively influence triage competency when professional feedback regarding triage performance is not provided [29]. In general, schools provide insufficient organizational resources. As most school nurses work alone at the schools, it is difficult for them to receive immediate feedback from peer experts.

Further, triage guidelines for schools have not yet been developed [10], and there is a lack of refresher training and continuing education for school nurses [10,30,31]. We found that the correlations between the Triage Competency Scale subdomains of Clinical Judgment and Expert Assessment and school nursing experience were the lowest in this study. This suggested that school nursing experience may not contribute sufficiently to the improved clinical judgment and expert assessment that account for a large proportion of a nurse’s overall triage competency level [16]. Therefore, developing an educational program focused on clinical judgment and expert assessment could be an effective way to improve triage competency among school nurses.

Previous studies reported that, at minimum, a triage nurse requires some clinical experience [27,28,29]; however, no clinical experience is required when hiring a school nurse [32]. Therefore, from the onset, most school nurses work alone in Korea [10,33]. We found that hospital nurse experience was a more important factor for triage competency than school nurse experience. Furthermore, nurses in the High Competency Group had a mean of 6.8 years of clinical experience. Thus, when hiring school nurses, it may be critical to consider hospital nurse experience to ensure an adequate triage competency level. For example, the hiring system method could be altered such that an extra credit is granted to school nurse applicants that have more than 6.8 years of hospital nurse experience.

In this study, the mean duration of experience as a school nurse in the Low Competency Groups (i.e., the lowest quartile of the Triage Competency Scale scores) was 8 years. To ensure patient safety, nurses with limited experience should not perform triage tasks in an unsupervised environment [29]. In addition, experienced nursing colleagues play an important role in developing competency and providing stability for less experienced nurses [27,34,35]. As school nurses work alone, existing systems must be improved to ensure that school nurses with low levels of triage competency can receive appropriate feedback and supervision. Further, to increase triage competency and reduce triage errors due to lack of experience, we propose that a system that allows less experienced school nurses to work with more experienced school nurses for their first 8 years as school nurses should be developed.

The 8 years of experience as a school nurse that were identified in this study is longer than the minimum experience required for triage nurses who work in hospitals. When working in an emergency room, the Emergency Nurses Association requires at least one year of experience [13], while another study recommended at least 2 years of experience [28]. One reason that more experience may be required for school nurses compared to their counterparts is that schools function as prehospital emergency practice sites. Compared to the hospital emergency room, the ability to collect and evaluate information promptly from an initial impression is a more notable requirement for the field triaging that is performed in a prehospital emergency field [36], and this ability is dependent on having a higher level of experience [26].

In the current study, serious emergency experience was an important influencing factor for triage competency. This finding was consistent with those from another study that demonstrated that emergency experience was associated with resuscitation competency [19]. Emergency experiences are actual situations where triage is applied. In particular, serious emergency experiences require familiarity with making quick decisions related to the patient’s life. These are practical situations in which triaging can be practiced under pressure [37]. From these experiences, school nurses can develop competency through self-evaluation and reflective practice [10,35]. Additionally, emergency experience positively influences competency development by increasing an individual’s motivation [38]. Therefore, educational programs that provide indirect experience in emergency situations, such as simulation exercises and case-based learning, could be effective scholastic methods for improving school nurses’ triage competency [10,27,39].

We found that the office location was also related to the triage competency, and school nurses who worked in metropolitan regions were more likely to be in higher triage competency groups than those who worked in non-metropolitan regions. Similarly, another study found that the triage accuracy rating scores of emergency room nurses who worked in urban hospitals were higher than those who worked in rural hospitals [21]. There are two explanations for this finding. First, there are differences in the environmental factors associated with professional development in metropolitan and non-metropolitan regions. Urban hospitals provide more educational opportunities to develop medical skills than non-urban hospitals [21]. Further, there are contextual factors related to the professional development of teachers [40]. In Korea, teachers who work in urban regions are more active in their professional development, such as completed education and degree achievements, than those who work in rural areas [41]. Second, the aforementioned finding may be related to differences in school size. According to the 2020 Korean Education Statistics [42], the average number of students in schools in metropolitan regions was 377, and this was notably higher than the average number of students in schools in non-metropolitan regions, which was 238. The frequency of emergencies increases with the number of students [43]. Therefore, school nurses working in metropolitan regions may experience more emergencies than those working in non-metropolitan regions, influencing their triage competency.

### Limitations

The current study had several limitations. First, we demonstrated a relationship between triage competency and school nurse experience, in addition to the respondents’ personal factors, such as age and education level. However, other personal factors that were explored in a previous study [44], including professionalism, critical thinking, confidence, and health status, were not included in the analysis. The organizational factors that may be associated with triage competency, such as school size, number of students visiting the health office, and other available resources, were also not included in the analysis. Future studies should adopt an integrated approach to the analysis of the factors related to triage competency by including organizational- and individual-level factors. Second, the level of nursing competency required to develop triage competency may differ based on the type of unit that the nurses worked in previously (i.e., emergency room vs. operation room). An analysis that takes into account both year of work experience and type of units in which nurses worked previously may provide insight into the practical implications of developing triage competency. However, the variable about specific hospital units was not collected in the current study. Further research is needed to include and analyze the differences in triage competency while considering the type of unit that the nurses worked in previously. Third, by analyzing the differences in the triage competency groups, we suggested primary standards that should be required for a school nurse to secure a certain level of triage competency. However, criteria based on triage accuracy may prove more useful in determining the minimum experience required to ensure the safety of individuals in schools. Thus, the relationship between triage accuracy and school nurse experience should be analyzed to examine which of the two factors would be considered a more important requirement. Finally, due to the convenience sampling method used in this study, the generalizability of the findings to other places or individuals may be limited.

## 5. Conclusions

In the current study, hospital nurse experience prior to working as a school nurse had higher odds of increasing triage competency than school nurse experience. Additionally, the mean duration of clinical experience for nurses in the High Competency Group was 6.8 years. These findings suggest that the hiring system for school nurses should consider previous hospital work experience when evaluating nurses. Moreover, the findings highlight the importance of establishing organizational support systems, such as guidelines and feedback systems, to improve triage competency. In particular, we propose the introduction of a policy that allows school nurses with minimal work experience, such as those who have less than 8 years of work experience (the mean duration of school nurse experience in the Low Competency Group), to work under appropriate supervision for a certain period of time.

In this study, we found that serious emergency experience and school location were factors that were associated with the school nurses’ triage competency. These findings demonstrate that a case-based education program that can provide simulations of emergency situations could be beneficial for school nurses who are employed in non-metropolitan regions and for those with limited emergency care experience in schools.

## Figures and Tables

**Table 1:** Differences in triage competency with respect to the participants’ general characteristics and work experience (*n* = 386).

Characteristics	All				Triage Competency Group				
					Low Group (*n* = 94)	Middle Group (*n* = 191)	High Group (*n* = 101)	χ^2^ or *F*	*p*
	*n* (%)	*M* (*SD*)	*t* or *F*	*p*	*n* (%) or *M* (*SD*)	*n* (%) or *M* (*SD*)	*n* (%) or *M* (*SD*)		
Education Level			6.99	<0.001				7.50	0.024
Bachelor’s Degree	309 (80.1)	82.18 (17.58)			84 (89.4)	150 (78.5)	75 (74.3)		
≥Master’s Degree	77 (19.9)	85.54 (18.06)			10 (10.6)	41 (21.5)	26 (25.7)		
Type of School			0.07	0.929				0.93	0.920
Elementary School	210 (54.4)	89.65 (17.90)			50 (53.2)	107 (56)	53 (52.5)		
Middle School	105 (27.2)	88.90 (20.57)			28 (29.8)	48 (25.1)	29 (28.7)		
High School	71 (18.4)	89.89 (19.23)			16 (17.0)	36 (18.8)	19 (18.8)		
School Location			8.44	0.003				7.89	0.019
Non-Metropolitan	150 (38.9)	86.03 (20.55)			48 (51.1)	68 (35.6)	34 (33.7)		
Metropolitan	236 (61.1)	91.69 (17.38)			46 (48.9)	123 (64.4)	67 (66.3)		
Serious Emergency Experience			3.93	<0.001				11.21	0.004
Yes	162 (42.0)	93.84 (18.50)			28 (29.8)	80 (41.9)	54 (53.5)		
No	224 (58.0)	86.34 (18.51)			66 (70.2)	111 (58.1)	47 (46.5)		
Emergency Nursing Care Certification			2.65	0.009				6.48	0.039
Yes	88 (22.8)	94.12 (17.79)			13 (13.8)	46 (24.1)	29 (28.7)		
No	298 (77.2)	88.12 (18.97)			81 (86.2)	145 (75.9)	72 (71.3)		
Age (years)		41.09 (10.08)			37.66 (10.05)	41.01 (9.91)	44.43 (9.41)	23.20	<0.001 L < M <H
* School Nurse Experience (years)		10.28 (9.80)			7.95 (9.92)	10.89 (9.83)	11.31 (9.39)	5.64	0.018 L < M, H
* Hospital Nurse Experience (years)		4.80 (4.38)			3.78 (3.33)	4.23 (3.77)	6.82 (5.56)	25.64	<0.001 L M < H

* Post hoc test according to the least significant difference test; L = Low Competency Group, lowest quartile (25%) of the total triage competency scores (≤78); M = Middle Competency Group, the middle 50% of the total triage competency scores (79–103); and H = High Competency Group, highest quartile (25%) of the total triage competency scores (>103). Abbreviations: M, mean; SD, standard deviation.

**Table 2:** Correlations between the triage competency subdomains and work experience.

	School Nurse Experience (*rho*, Years)	Hospital Nurse Experience (*rho*, Years)
Clinical Judgment	0.14 **	0.25 ***
Expert Assessment	0.16 **	0.20 ***
Management of Medical Resources	0.22 ***	0.18 ***
Timely Decisions	0.20 ***	0.26 ***
Communication	0.20 ***	0.18 ***
Total score	0.19 ***	0.25 ***

** *p* < 0.01; *** *p* < 0.001.

**Table 3:** Ordinal logistic regression analysis results for predicting factors related to triage competency (*n* = 386).

	*B*	Adjusted Odds Ratio	95% CI	*p*-Value
Education level				
Bachelor’s degree	Ref			
≥Master’s degree	0.19	1.21	0.72–2.04	0.472
School Location				
Non-Metropolitan	Ref			
Metropolitan	0.49	1.63	1.09–2.45	0.017
Serious Emergency Experience				
No	Ref			
Yes	0.56	1.76	1.16–2.65	0.008
Emergency Nursing Care Certification				
No	Ref			
Yes	0.45	1.56	0.97–2.51	0.066
School Nurse Experience (years)	0.02	1.02	1.00–1.05	0.037
Hospital Nurse Experience (years)	0.13	1.14	1.09–1.20	<0.001
Likelihood: χ*²*(*df*) = 56.34(6), *p* < 0.001; Brant test of parallelism: χ*²*(*df*) = 8.73(6), *p* = 0.19; Cox & Snell = 0.14, Nagelkerke *R*^2^ = 0.16, McFadden *R*^2^ = 0.07.				

Abbreviations: CI, confidence interval.

## Data Availability

Not applicable.
